# Quality of life in patients at first time visit for sleep disorders of breathing at a sleep centre

**DOI:** 10.1186/1477-7525-11-207

**Published:** 2013-12-12

**Authors:** Serena Iacono Isidoro, Adriana Salvaggio, Anna Lo Bue, Salvatore Romano, Oreste Marrone, Giuseppe Insalaco

**Affiliations:** 1 National Research Council of Italy, Institute of Biomedicine and Molecular Immunology “A. Monroy”, Via Ugo La Malfa, 153 - 90146, Palermo, Italy

**Keywords:** OSA, Quality of life, Excessive daytime sleepiness, Obesity, Hypoxemia, Gender

## Abstract

**Introduction:**

Sleep-disordered breathing adversely affects daytime alertness and cognition. Obstructive sleep apnea (OSA) patients have several typical symptoms including habitual snoring, excessive daytime sleepiness, fatigue, lack of concentration, memory impairment, and at times psychological disturbances. We evaluated different aspects in the health related quality of life (HRQoL) in subjects referred to our sleep laboratory for their first examination for suspicion of OSA.

**Methods:**

One hundred ninety-eight consecutive outpatients (152 M) (mean age 52.7 ± 12.8 years, range 18–82 years; mean BMI 31.0 ± 6.5 kg/m^2^, range 17.3-57.8 kg/m^2^) were evaluated with two self-reported questionnaires for HRQoL assessment: Psychological General Well-Being Index (PGWBI), that asses anxiety, depressed mood, positive well-being, self-control, general health, vitality, and 12-Item Short-Form Health Survey (SF-12), consisting assesses of Physical and Mental Component Summaries (PCS and MCS). Epworth Sleepiness Scale (ESS) was used to assess daytime sleepiness before nocturnal diagnostic examination.

**Results:**

Subjects showed variable HRQoL scores. HRQoL was worse in women than men and it decreased with age. No relation was found with AHI severity (range 0–129 n/h). BMI and TSat_90_ (range 0–87.9%) affected physical health perception (SF-12 PCS). Furthermore TSat_90_ influenced PGWBI Vitality subscale. Subjects with ESS > 10 showed a worse HRQoL profile (p < 0.001) in SF-12 and in PGWBI. Multiple regression analysis showed that age, BMI and ESS were significant predictors of SF-12 PCS (p < 0.001; r^2^ = 0.23).

**Conclusions:**

A worse HRQoL perception among subjects referred for OSA suspicion was not related to disease severity. BMI and hypoxemia influenced only some HRQoL dimensions, while excessive daytime sleepiness worsens all HRQoL components considered.

## Introduction

Obstructive sleep apnea (OSA) is a common sleep disorder characterized by intermittent partial or complete upper airway obstruction during sleep, associated to intermittent hypoxemia, recurrent arousals, sleep fragmentation and poor sleep quality. The prevalence of OSA with accompanying daytime sleepiness is approximately 3 to 7% for adult men and 2 to 5% for adult women in the general population. Factors that increase susceptibility to the disorder include age, male sex, menopause, obesity, craniofacial abnormalities, family history, and health behaviours such as cigarette smoking and alcohol use [1].

Patients with OSA may present several typical symptoms including habitual snoring (often disruptive to bed partners), feeling of unrefreshed awaking, excessive daytime sleepiness (EDS), fatigue, lack of concentration, memory impairment, and at times psychological disturbances [2,3]. Although a relationship between the severity of sleep respiratory disorders and EDS has been observed, recent studies pointed out that this relationship is poor [4,5].

Symptoms of cognitive and emotional disorders are accompanied by cardiovascular impairment that may eventually lead to more serious conditions such as hypertension, arrhythmias, coronary artery disease or stroke [6].

Literature highlights how perceived well-being and Health Related Quality of Life (HRQoL) are deteriorated in sleep-disordered breathing, in particular OSA [7]. OSA patients often report a poor quality of life in social, emotional, and physical domains [8]; emotional disturbance in OSA may also give rise to family and social conflict [9,10].

In this study, in order to evaluate the perception of well-being in patients afferent to a laboratory for diagnosis and treatment of sleep-disordered breathing, questionnaires for non-specific pathologies were administered by an assistant psychologist before the first examination. Unspecific disease questionnaires were chosen to investigate psychological dimensions of HRQoL, as they were addressed to a population that had not yet received a specific diagnosis.

For this purpose we used the *Psychological General Well-Being Index* (PGWBI), a validated HRQoL measure, widely used in epidemiological research to provide a general evaluation of self-perceived psychological health and well-being [11], and extensively used to record well-being in different patient populations. As far as we know PGWBI has never been administered in OSA population. PGWBI addresses the impact of symptoms on well-being and is applicable both for healthy and patient populations [12]. It focuses on self-representations of psychological general well-being, reflecting subjective well-being or distress. We administered also the *12-Item Short-Form Health Survey* (SF-12), one of the most widely used instruments to measure HRQoL and monitor health in general and in specific populations. SF-12 is a multipurpose generic measure for all ages or disease groups [13]. This tool was administered with PGWBI to guarantee the possibility to further expand the dimensions of wellness with a comparison and control measure of HRQoL; indeed the validity of SF-12 has been also evaluated in patients with sleep apnea under CPAP treatment, showing results identical to those of the SF-36 [14].

The aims of this study were to evaluate self-perceived psychological HRQoL in patients pertaining to a laboratory for sleep related breathing disorders and to verify which features of OSA (such as obesity, disease severity, nocturnal hypoxia, EDS) might contribute significantly to subjective well-being, and which dimensions of the latter may suffer greater damage.

## Methods

We performed a study involving 198 consecutive outpatients (46 F, 152 M), between 18 and 82 years old (mean age 52.7 ± 12.8 yrs), afferent to our Sleep Laboratory. Patients competent to sign informed consent and willing to participate in the study were included. Patients with a prior diagnosis or treatment for OSAS were excluded, as were subjects who did not consent or did not complete full diagnostic process, or PGWBI and SF-12 questionnaires. Patients affected by psychiatric and neurological diseases were also excluded. Subjects underwent a detailed evaluation that included clinical history focused on sleep-related symptoms. In the sample, 12 subjects presented an ischemic heart disease, 7 subjects had an ischemic cardiovascular disease and 15 subjects were diabetics. Body mass index (BMI) was calculated. The ethical committee of our Institution authorised anonymous scientific utilisation of data collected for routine clinical work.

Nocturnal monitoring was performed with a portable computerized system for OSA diagnosis (Somté Compumedics Inc.; Abbotsford, VIC, Australia). The recorded signals were airflow, snoring, thoracic effort, abdominal effort, limb movement, body position, electrocardiogram, arterial oxygen saturation, pulse rate, and pulse waveform. Duration of recordings was at least 6 h. Apneas and hypopneas were visually scored. Apneas were defined as lack of airflow for at least 10 s. Hypopneas were defined as discernible reductions in airflow or thoraco-abdominal movements for at least 10 s followed by an arterial oxygen saturation fall >3% [15]. Apnea-hypopnea index (AHI) was calculated as number of apnoeas plus hypopneas per hour of estimated total sleep time. The definition of apneas and hypopneas followed the AASM standard criteria [15,16]. OSAS was considered mild if the AHI was ≥5 per hour and <15 per hour, as moderate if AHI was ≥15 per hour and ≤30 per hour and as severe if AHI was >30 per hour. Percent of the night with O_2_ saturation <90% (TSat_90_) was measured.

### Questionnaires

The *Psychological General Well-Being Index (PGWBI)*[17] was used to measure subjective mental health [18]. The responses to 22 questions are arranged in six subscales: anxiety (five items), depressed mood (three items), positive well-being (four items), self-control (three items), general health (three items) and vitality (four items). Item responses are rated on a six-point Likert scale ranging from 0 (highest possible distress) to 5 (completely healthy status). The six-scale scores were computed by summing the relevant items. Higher scores indicate better health. The six scales can be further summed to provide a global index score representing one comprehensive subjective well-being ranging from 0 to 110 [19]. A global score <60 suggests a severe distress; from 60 to 72 suggests a moderate distress; and >72 suggests a positive well-being.

*The 12-Item Short-Form Health Survey (SF-12)* is the shorter health self-administered questionnaire derived from the SF-36, allowing faster assessment of patients and producing physical and emotional component summaries without any substantial loss of information compared to the SF-36 [20]. Two subscales are derived from the SF-12: the Physical Component Summary (PCS) and the Mental Component Summary (MCS). The PCS includes questions about physical functioning, role limitations due to physical health problems, bodily pain and general health. The MCS includes questions about vitality (energy/fatigue), social functioning, role limitations due to emotional problems, and mental health (psychological distress and psychological well-being). The PCS and MCS are standardised to a mean of about 50, with a score above 50 representing better than average function and below 50 poorer than average function [21].

The *Epworth Sleepiness Scale (ESS)* was used to assess daytime sleepiness. Patients rated the likelihood of falling asleep in eight specific situations on a 0–3 scale, with 0 meaning no chance at all of falling asleep, and 3 representing a high chance of falling asleep. Thus, the scale goes from 0 to 24. A score > 10 suggests excessive daytime sleepiness [22].

### Statistical analysis

Difference between means was assessed by the non-parametric Wilcoxon test. Relationships between selected variables were identified through simple linear regression and multiple linear regression. Data were reported as mean ± SD. A p < 0.05 was considered significant. Statistical analysis was performed by commercial software (JMP 8.0 SAS Institute Inc.).

## Results

Characteristics of participants to the study, questionnaires scores and nocturnal polygraphic results are reported in Table 1. The mean of the scores was lower than reference data in both HRQoL questionnaires [11,13]. A significant relationship was found between age and all questionnaires dimensions, except for PGWBI Anxiety and Vitality subscales (Table 2). Dividing the sample by gender, male subjects (n = 152) reported higher scores compared to females (n = 46) in PGWBI, except for Anxiety and Vitality subscales, and in both SF-12 summaries (Table 3). No differences were found in BMI, AHI, TSat_90_%, ESS and age between men and women.

**Table 1:** Summary of patient participants to the study characteristics, questionnaires score and nocturnal polygraphic results

**Variables**	**Values**	
N	198 (152 M - 46 F)	
Age (yr)	52.7 ± 12.8	(18 – 82)
BMI (kg/m^2^)	31.0 ± 6.5	(17.3 - 57.8)
ESS score	8.8 ± 5.2	(0 - 24)
PGWBI	70.9 ± 16.0	(20 – 101)
Anxiety	15.7 ± 4.6	(1 - 25)
Depression	12.1 ± 2.7	(0 - 15)
Well-being	11.4 ± 3.7	(3 - 19)
Self-control	11.0 ± 2.7	(0 - 15)
Health	9.6 ± 2.7	(2 - 15)
Vitality	11.2 ± 3.8	(0 - 19)
SF-12 PCS	44.6 ± 9.2	(20.2 - 63.8)
SF-12 MCS	45.8 ± 10.9	(16.7 - 66.3)
AHI (n/h)	33.6 ± 27.6	(0 - 129)
TSat_90_ (%)	15.1 ± 21.8	(0 - 87.9)

Values are mean ± SD (range). BMI, body mass index; ESS, Epworth Sleepiness Scale; PGWBI, Psychological General Well-Being Index; SF-12, 12-Item Short-Form Health Survey; PCS, Physical Component Summary; MCS, Mental Component Summary; AHI, Apnea hypopnea index; TSat_90_, percent of the night at less than 90% oxygen saturation.

**Table 2:** Participants characteristics and nocturnal polygraphic results vs health-related quality of life

	**PGWBI**							**SF-12**	
	**TOT**	**ANX**	**DEP**	**WB**	**SC**	**HEA**	**VIT**	**PCS**	**MCS**
**Age**	p = 0.004	*NS*	p < 0.001	p < 0.001	p = 0.008	p = 0.007	*NS*	p < 0.001	*NS*
**BMI**	*NS*	*NS*	*NS*	*NS*	*NS*	*NS*	*NS*	p < 0.001	*NS*
**AHI**	*NS*	*NS*	*NS*	*NS*	*NS*	*NS*	*NS*	p = 0.010	*NS*
**TSat**_**90**_	*NS*	*NS*	*NS*	*NS*	*NS*	*NS*	p < 0.001	p < 0.001	*NS*
**ESS**	p < 0.001	p < 0.001	p = 0.003	p = 0.011	p < 0.001	p < 0.001	p < 0.001	p < 0.001	p = 0.003

PGWBI, Psychological General Well-Being Index; TOT, Total score; ANX, Anxiety subscale; DEP, Depression subscale; WB, Well-Being subscale; SC, Self-Control subscale; HEA, Health subscale; VIT, Vitality subscale; SF-12, 12-Item Short-Form Health Survey; PCS, Physical Component Summary; MCS, Mental Component Summary; BMI, body mass index; AHI, Apnea hypopnea index; TSat_90_, percent of the night at less than 90% oxygen saturation; ESS, Epworth Sleepiness Scale.

**Table 3:** Gender differences on health-related quality of life

	**PGWBI**							**SF-12**	
	**TOT**	**ANX**	**DEP**	**WB**	**SC**	**HEA**	**VIT**	**PCS**	**MCS**
**Female (n = 46)**	64.8 ± 18.5	14.5 ± 5.9	11.1 ± 3.3	9.8 ± 3.8	10.3 ± 2.9	8.7 ± 2.3	10.3 ± 3.9	42.0 ± 9.6	42.1 ± 12.1
**Male (n = 151)**	72.7 ± 15.1	16.1 ± 4.1	12.3 ± 2.5	11.9 ± 3.5	11.2 ± 2.7	9.9 ± 2.7	11.4 ± 3.7	45.3 ± 9.0	46.8 ± 10.2
**p value**	0.007	*NS*	0.030	<0.001	0.042	0.010	*NS*	0.049	0.019

Values are mean ± SD (range). PGWBI, Psychological General Well-Being Index; TOT, Total score; ANX, Anxiety subscale; DEP, Depression subscale; WB, Well-Being subscale; SC, Self-Control subscale; HEA, Health subscale; VIT, Vitality subscale; SF-12, 12-Item Short-Form Health Survey; PCS, Physical Component Summary; MCS, Mental Component Summary.

BMI was linearly correlated with PCS (p < 0.001) (Table 2). Subdividing the sample into two classes of BMI (BMI < 30 n = 98, BMI ≥ 30 n = 100), lower scores in the SF-12 PCS were highlighted in obese patients (10.6 ± 3.8) compared to subjects with BMI < 30 (47.1 ± 7.6; p < 0.001), but no significant difference was found in SF-12 MCS and in PGWBI.

AHI was inversely related to SF-12 PCS (p = 0.010), but not with PGWBI, total and subscales (Table 2). Even splitting the AHI by severity (AHI < 5 n = 30, 5 ≤ AHI ≤ 15 n = 37, 15 < AHI < 30 n = 41, AHI ≥ 30 n = 90) there were no differences in HRQoL dimensions.

TSat_90_ was linearly related with PGWBI Vitality subscale (p = 0.006) and SF-12 PCS (p = 0.004); increasing TSat_90_ was associated with a worsening of the vitality and perceived physical health (Table 2). Subjects with TSat_90_ > 30% (n = 38) as compared to those with TSat_90_ ≤ 30% (n = 160) obtained lower scores in PGWBI Vitality subscale (9.6±3.9 vs 11.5 ± 3.7, p = 0.005) and in SF-12 PCS (41.7 ± 9.3 vs 45.2 ± 9.1, p = 0.029).

Of 198 subjects, 62 (31.3%) had an ESS > 10; no correlation was found between age and ESS. ESS showed an inverse linear relationship with the scores of all PGWBI subscales as well as with SF-12 summaries (p < 0.010). The subjects with EDS (ESS > 10) had lower scores in all PGWBI subscales and in SF-12 summaries (Table 4).

**Table 4:** Health-related quality of life data on Epworth Sleepiness Scale

	**ESS ≤10**	**ESS >10**	**p value**
	**(n = 136)**	**(n = 62)**	
**PGWBI**	74.7 ± 14.6	62.6 ± 16.8	p < 0.001
**Anxiety**	16.5 ± 4.1	13.9 ± 5.1	p = 0.002
**Depression**	12.6 ± 2.3	10.9 ± 3.2	p < 0.001
**Well-being**	11.9 ± 3.6	10.2 ± 3.7	p = 0.003
**Self-control**	11.5 ± 2.4	9.9 ± 3.1	p < 0.001
**Health**	10.1 ± 2.6	8.4 ± 2.5	p < 0.001
**Vitality**	12.0 ± 3.7	9.2 ± 3.2	p < 0.001
**SF-12 PCS**	46.4 ± 8.3	40.4 ± 9.9	p < 0.001
**SF-12 MCS**	47.5 ± 10.3	41.9 ± 11.1	p < 0.001

Values are mean ± SD (range). ESS, Epworth Sleepiness Scale; PGWBI, Psychological General Well-Being Index; SF-12, 12-Item Short-Form Health Survey; PCS, Physical Component Summary; MCS, Mental Component Summary.

Multiple regression analysis showed that significant predictors of SF-12 PCS were age, BMI and ESS (p < 0.001; r^2^ = 0.23).

## Discussion

The results of this study confirmed that OSA has an impact on patients HRQoL [23]. As concerns gender and age differences, women had significantly lower scores than men and there were differences related to age. Similar results have been shown with other HRQoL instruments [18]. Analysis of the relationships between BMI or TSat_90_ and the questionnaires results showed that overweight, obesity and hypoxia negatively affect physical health perception as assessed by the SF-12 PCS. Furthermore TSat_90_ influenced also the PGWBI Vitality subscale, while no relationship was found between AHI severity and HRQoL as assessed by questionnaires applied. In all PGWBI dimensions and in SF-12 PCS and MCS the patients with excessive daytime sleepiness did score significantly worse than the patients with ESS < 10, irrespective of OSA severity. It thus appears that in our sample, the perception of their psychological well-being is not influenced by the severity of disease (AHI), and that overweight and obesity, as well as hypoxemia, influence the perception of the energy and physical health.

Women showed a worse HRQoL than men. Similar results were observed in many other studies, despite they adopted other HRQoL or mood evaluation instruments [24,25]. Women in a healthy population report poorer well-being and have a higher symptomatic complaint rate [12]. Gender differences could be explained by women characteristics such as greater bodily attention, generalized psychological disturbance, as well as social acceptance for women to express distress [26]. As reported by the manuals of the tools used in our study, the HRQoL decreases with age [11,13]. However, in our sample neither gender nor age were related to any aspect of HRQoL. Further studies are needed to confirm their lack of influence on the PGWBI Vitality and Anxiety subscales.

Body weight-related issues are common among OSA patients. In our sample, patients with BMI ≥ 30 showed significantly worse scores in SF-12 PCS, representing a bad physical health perception.

Unlike BMI, AHI was not related with the previously mentioned dimension of HRQoL. An influence of BMI on patients well-being, at least partly independent of the severity of sleep respiratory disorders, has already been pointed out in other studies: for example, Resta et al. [4] documented that obesity is associated with hypersomnolence even in absence of sleep-disordered breathing. Similarly Lacasse et al. [10] found a weak correlation between impairment of HRQoL and OSA severity, while other studies did not observe any correlation between increase in severity illness and HRQoL burden [8,25]. Conversely, in apparent contrast with our results, a recent study found that severe sleep apnea (RDI > 30) was associated with reduction in general health perception, mental health, vitality and social functioning [27]; this discrepancy could be due to the difference in population studied since the authors excluded from the sample subjects with an AHI <5. Our goal was to assess the subjects at first time visit: this allowed us to estimate also HRQoL of no-OSA subjects, reporting a wide range of symptoms and disease severity that led them to consult a sleep laboratory.

As regards nocturnal hypoxia, to our knowledge few studies have investigated its impact on HRQoL, particularly in OSA patients. A recent study demonstrated an impact of sleep disruption and hypoxemia in OSA on mood [28]. A possible influence of nocturnal hypoxia on HRQoL was assessed in patients with COPD. In this regard there have been conflicting results [29] but the effects of hypoxia were often regarded as negligible. This is in agreement with our results, which show a significantly negative correlation of nocturnal hypoxia only with perception of energy (PGWBI Vitality subscale) and physical health (SF-12 PCS). Thus, it may be speculated that hypoxia negatively influences perception of physical power and could contribute to restrict many activities.

Naismith et al. [30] pointed out that in OSA the severity of depression and anxiety appear to be related more to the degree of daytime sleepiness than of nocturnal hypoxemia. Similarly in another study [31] difficulty initiating or maintaining sleep and excessive sleepiness did predict widespread disturbance in quality of life measures. Our data show that the HRQoL perception worsened with increasing sleepiness since subjects with ESS > 10 obtained worse scores in PGWBI questionnaire and in both SF-12 summaries. This result suggests that, unlike the other parameters taken into account in this study, EDS affects all the dimensions of HRQoL analyzed by both questionnaires. Similar results were found by Dodel et al. [32], in a study on narcoleptic patients, and by Jacobsen et al. [33], who evaluated factors associated to EDS in OSA patients. Among the variables evaluated in this study, EDS was the most strongly related to the various dimensions of general HRQoL and psychological well-being, as the emotional functioning and the interpersonal relationship, although it may not be their only determinant. Further studies could explore the possible role of other factors as determinants of HRQoL in OSA. Besides, they could further investigate which dimensions of HRQoL are most affected by the disease [10]. Bixler et al. [5] showed that the prevalence of EDS was higher in the young and the very old, the former most likely a result of increased unmet sleep needs and the latter due to increased health problems and medical illness. In our sample we found no significant correlation between age and EDS. This difference could be due to the age of the sample, since in Bixler’s study older subjects were included.

As far as we know, so far influences of gender, BMI, ESS on SF-12 outcomes have not been examined at the same time in other studies. However, other studies separately showed the influence of each of them on HRQoL. A survey of primary care patients by Finkelstein [34] found that SF-12 PCS markedly decreased with BMI above the normal weight range. Obesity is a factor consistently linked to daytime sleepiness, with obese subjects twice as likely to report EDS than non-obese individuals [35]. EDS influences more or less all aspects of life to such an extent that somnolent people perceive themselves as being generally more limited by their health condition than those without it [36]. Therefore it could be possible to affirm that the combination of these factors worsens the general physical health perception evaluated by SF-12.

Our study also has some limitations, the most important being related to the unavailabity of sleep EEG data since nocturnal monitoring was performed with unattended poligraphy. However estimation of total sleep time was performed by excluding recording epochs with a clear awakening. Furthermore, because of the different size of the male and females subgroups, HRQoL differences could be attributed to a lack of statistical power. This difference between males and females HRQoL requires further studies.

## Conclusions

In conclusion, the available data confirm that even mild OSA is associated with impairment in HRQoL [30]. Our study shows that disease severity is not related to HRQoL perception. BMI affects physical health perception as evaluated by SF-12 PCS, and hypoxemia influences the latter HRQoL dimension and Vitality (PGWBI subscale). HRQoL among subjects referred for suspicion of OSA is mostly related to subjective excessive daytime sleepiness which in our sample, as well as in other studies, is not always present in OSA subjects. Sleep disorders have a great impact on HRQoL because they impair an individual normal function, mood, and well-being, making their assessment of paramount importance to patients. PGWBI questionnaire, used for the first time in OSA population to our knowledge, proved to be a useful tool to analyze which dimensions of HRQoL are most impaired in a population afferent to a sleep lab. Our sample presents slightly lower scores than the averages of the general population reported by the manuals of the questionnaires [13,15]. Thus, from a clinical point of view, our data reinforce the importance of investigating HRQoL in patients afferent to a sleep lab, even in those without OSAS. HRQoL therefore appears necessary for a complete assessment of patients and to program specific individualized interventions to improve well-being.

## Competing interests

The authors declare that they have no competing interests. The authors have no conflict of interest associated with this publication.

## Authors’ contributions

Dr. II was responsible for collection of all retrospective and follow-up data and for organizing the data base. Dr. S conceived the study, contributed to design the study, recorded baseline data and contributed to draft the manuscript. Dr. LB recorded baseline data and contributed for organizing the data base and to draft the manuscript. Dr. M recorded baseline data, contributed to design the study and to draft the manuscript. Dr. R performed the statistical analysis and contributed to the interpretation of the data. Dr. I conceived the study, contributed to the study design, recorded baseline data and drafted the manuscript. All authors actively discussed the subject, revised the paper, and provided final approval.

## References

[B1] Punjabi NM The epidemiology of adult obstructive sleep apnea Proc Am Thorac Soc 2008 11 136 143 10.1513/pats.200709-155MG 18250205 PMC2645248

[B2] Flemons WW Clinical practice. Obstructive sleep apnea N Engl J Med 2002 11 498 504 10.1056/NEJMcp012849 12181405

[B3] Kales A Caldwell A Cadieux R Vela-Beuno A Ruch LG Mayes SD Severe obstructive sleep apnea-II: associated psychological and psychosocial consequences J Chronic Dis 1985 11 426 437 10.1016/0021-9681(85)90138-9 3998057

[B4] Resta O Foschino Barbaro MP Bonfitto P Giliberti T Depalo A Pannacciulli N De Pergola G Low sleep quality and daytime sleepiness in obese patients without obstructive sleep apnoea syndrome J Intern Med 2003 11 536 543 10.1046/j.1365-2796.2003.01133.x 12702031

[B5] Bixler EO Vgontzas AN Lin H-M Calhoun SL Vela-Bueno A Kales A Sleepiness in a general population sample: the role of sleep apnea, age, obesity, diabetes, and depression J Clin Endocrinol Metab 2005 11 4510 4515 10.1210/jc.2005-0035 15941867

[B6] Shahar E Whitney CW Redline S Lee ET Newman AB Nieto FJ O'Connor GT Boland LL Schwartz JE Samet JM Sleep-disordered breathing and cardiovascular disease: cross-sectional results of the Sleep Heart Health Study Am J Respir Crit Care Med 2001 11 19 25 10.1164/ajrccm.163.1.2001008 11208620

[B7] Flemons WW Reimer MA Measurement properties of the Calgary sleep apnea quality of life index Am J Respir Crit Care Med 2002 11 159 164 10.1164/ajrccm.165.2.2010008 11790647

[B8] D'Ambrosio C Bowman T Mohsenin V Quality of life in patients with obstructive sleep apnea: effect of nasal continuous positive airway pressure - a prospective study Chest 1999 11 123 129 10.1378/chest.115.1.123 9925072

[B9] Pillar G Lavie P Psychiatric symptoms in sleep apnea syndrome: effects of gender and respiratory disturbance index Chest 1998 11 697 703 10.1378/chest.114.3.697 9743153

[B10] Lacasse Y Godbout C Sériès F Health-related quality of life in obstructive sleep apnoea Eur Respir J 2002 11 499 503 10.1183/09031936.02.00216902 11936529

[B11] Grossi E Groth N Mosconi P Cerutti R Pace F Compare A Apolone G Development and validation of the short version of the Psychological General Well-Being Index (PGWB-S) Health Qual Life Outcomes 2006 11 88 10.1186/1477-7525-4-88 17105655 PMC1647268

[B12] Sjöland H Wiklund I Caidahl K Hartford M Karlsson T Herlitz J Improvement in quality of life differs between women and men after coronary artery bypass surgery J Intern Med 1999 11 445 454 10.1046/j.1365-2796.1999.00500.x 10363744

[B13] Kodraliu G Mosconi P Groth N Carmosino G Perilli A Gianicolo EA Rossi C Apolone G Subjective health status assessment: evaluation of the Italian version of the SF-12 Health Survey. Results from the MiOS Project J Epidemiol Biostat 2001 11 305 316 10.1080/135952201317080715 11437095

[B14] Jenkinson C Layte R Jenkinson D Lawrence K Petersen S Paice C Stradling J A shorter form health survey: can the SF-12 replicate results from the SF-36 in longitudinal studies? J Public Health Med 1997 11 179 186 10.1093/oxfordjournals.pubmed.a024606 9243433

[B15] American Academy of Sleep Medicine Task Force Report Sleep-related breathing disorders in adults: recommendations for syndrome definition and measurement techniques in clinical research Sleep 1999 11 667 689 10450601

[B16] Collop NA Anderson WM Boehlecke B Claman D Goldberg R Gottlieb DJ Hudgel D Sateia M Schwab R Portable Monitoring Task Force of the American Academy of Sleep Medicine Clinical guidelines for the use of unattended portable monitors in the diagnosis of obstructive sleep apnea in adult patients. Portable monitoring task force of the American Academy of Sleep Medicine J Clin Sleep Med 2007 11 737 747 18198809 PMC2556918

[B17] Grossi E Mosconi P Groth N Niero M Apolone G Il Questionario Psychological General Well Being. Questionario per la valutazione dello stato generale di benessere psicologico. Versione Italiana 2002 Milan: Edizioni Mario Negri

[B18] Dupuy H Wenger NK, Mattson ME, Furburg CD, Elinson J The Psychological General Well-Being (PGWB) index Assessment of Quality of Life in Clinical Trials of Cardiovascular Therapies 1984 New York: Le Jacq Publishing 170 183

[B19] Serpentini S Del Bianco P Alducci E Toppan P Ferretti F Folin M De Salvo GL Nitti D Pucciarelli S Psychological well-being outcomes in disease-free survivors of mid-low rectal cancer following curative surgery Psychooncology 2011 11 706 714 10.1002/pon.1763 20878856

[B20] Gandek B Ware JE Aaronson NK Apolone G Bjorner JB Brazier JE Bullinger M Kaasa S Leplege A Prieto L Sullivan M Cross-validation of item selection and scoring for the SF-12 Health Survey in nine countries: results from the IQOLA Project. International Quality of Life Assessment J Clin Epidemiol 1998 11 1171 1178 10.1016/S0895-4356(98)00109-7 9817135

[B21] Ware JE Kosinski M Turner-Bowker DM Gandeck B User’s Manual for the SF-12v2 Health Survey (With a Supplement Documenting SF-12 Health Survey) 2007 Quality Metric Incorporated: Lincoln, RI

[B22] Johns MW A new method for measuring daytime sleepiness: the Epworth sleepiness scale Sleep 1991 11 540 545 1798888 10.1093/sleep/14.6.540

[B23] Pichel F Zamarrón C Magán F del Campo F Alvarez-Sala R Suarez JR Health-related quality of life in patients with obstructive sleep apnea: effects of long-term positive airway pressure treatment Respir Med 2004 11 968 976 10.1016/j.rmed.2004.03.009 15481273

[B24] Ye L Pien GW Ratcliffe SJ Weaver TE Gender differences in obstructive sleep apnea and treatment response to continuous positive airway pressure J Clin Sleep Med 2009 11 512 518 20465016 PMC2792965

[B25] Moore P Wayne AB Ancoli-Israel S Dimsdale JE Association between polysomnographic sleep measures and health-related quality of life in obstructive sleep apnea J Sleep Res 2001 11 303 308 10.1046/j.1365-2869.2001.00264.x 11903860

[B26] Barsky AJ Peekna HM Borus JF Somatic symptom reporting in women and men J Gen Intern Med 2001 11 266 275 10.1046/j.1525-1497.2001.016004266.x 11318929 PMC1495200

[B27] Karkoulias K Lykouras D Samsonas F Karaivazoglou K Sargianou M Drakatos P Spiropoulos K Assimakopoulos K The impact of obstructive sleep apnea syndrome severity on physical performance and mental health. The use of SF-36 questionnaire in sleep apnea Eu Rev Med Pharmacol Sci 2013 11 531 536 23467954

[B28] El-Ad B Lavie P Effect of sleep apnea on cognition and mood Int Rev Psychiatry 2005 11 277 282 10.1080/09540260500104508 16194800

[B29] Sant’Anna Ferreira CA Stelmach R Zanetti Feltin MI Filho WJ Chiba T Cukier A Evaluation of health related quality of life in low income patients with COPD receiving long term oxygen therapy Chest 2003 11 136 141 10.1378/chest.123.1.136 12527614

[B30] Naismith S Winter V Gotsopoulos H Hickie I Cistulli P Neurobehavioral functioning in obstructive sleep apnea: Differential effects of sleep quality, hypoxemia and subjective sleepiness J Clin Exp Neuropsychol 2004 11 43 54 10.1076/jcen.26.1.43.23929 14972693

[B31] Sateia MJ Neuropsychological impairment and quality of life in obstructive sleep apnea Clin Chest Med 2003 11 249 259 10.1016/S0272-5231(03)00014-5 12800782

[B32] Dodel R Peter H Spottke A Noelker C Althaus A Siebert U Walbert T Kesper K Becker HF Mayer G Health-related quality of life in patients with narcolepsy Sleep Med 2007 11 733 741 10.1016/j.sleep.2006.10.010 17512797

[B33] Jacobsen JH Shi L Mokhlesi B Factors associated with excessive daytime sleepiness in patients with severe obstructive sleep apnea Sleep Breath 2013 11 629 635 10.1007/s11325-012-0733-z 22733531

[B34] Finkelstein MM Body mass index and quality of life in a survey of primary care patients J Fam Pract 2000 11 734 737 10947141

[B35] Slater G Steier J Excessive daytime sleepiness in sleep disorders J Thorac Dis 2012 11 608 616 23205286 10.3978/j.issn.2072-1439.2012.10.07 PMC3506799

[B36] Wu S Wang R Ma X Zhao Y Yan X He J Excessive daytime sleepiness assessed by the Epworth Sleepiness Scale and its association with health related quality of life: a population-based study in China BMC Public Health 2012 11 849 10.1186/1471-2458-12-849 23039935 PMC3524046

